# Global, regional and national burden of colon and rectum cancer attributable to high fasting plasma glucose: a systematic analysis for the Global Burden of Disease Study 2021

**DOI:** 10.3389/fonc.2026.1690349

**Published:** 2026-02-27

**Authors:** Ke Lu, Gang Xu

**Affiliations:** 1 Department of General Surgery, Hangzhou Xixi Hospital, Hangzhou Sixth People’s Hospital, Hangzhou Xixi Hospital Affiliated to Zhejiang Chinese Medical University, Hangzhou, China; 2 Department of General Surgery, Zhejiang Hospital, Hangzhou, China

**Keywords:** colorectal cancer, DALYs, Global Burden of Disease 2021, health inequality, high fasting plasma glucose, mortality

## Abstract

**Background:**

Colorectal cancer (CRC), the third most common malignant cancer globally, imposes a substantial public health burden. Emerging evidence has highlighted hyperglycemia as an independent risk factor for CRC. Estimates of the global burden of high fasting plasma glucose (FPG)-derived CRC are lacking.

**Methods:**

We used the Global Burden of Disease (GBD) 2021 data to analyze deaths and disability-adjusted life years (DALYs) of CRC attributable to high FPG across 204 countries or territories. The GBD standard population was used to calculate age-standardized rates. Joinpoint regression identified temporal trends (1990–2021), and Bayesian age-period-cohort modeling projected future burdens (2022–2036). Health inequalities were assessed by stratifying the analyses according to the Sociodemographic Index (SDI).

**Results:**

Globally, high FPG-related CRC deaths increased from 31,907 (1990) to 82,421 (2021), with DALYs loss rising from 715,716 to 1,750,923. Age-standardized mortality rates (ASMR) and DALY rates (ASDR) increased by an average of 0.31% overall during the study period but declined post-2019. High SDI regions bore the highest burden (ASMR: 1.33 per 100,000 person-years; ASDR: 27.65 per 100,000 person-years), yet trends stabilized, whereas low-middle SDI regions saw the sharpest rise (ASMR average annual percentage change [AAPC]: 1.82%; ASDR AAPC: 1.77%). Males had a higher burden of CRC attributable to high FPG than females. Health inequality analysis showed that absolute differences in DALYs slightly increased (slope index of inequality of DALYs increased from 20.251 to 20.292), while relative differences decreased (concentration index of DALYs improvement: −0.344 to −0.294). Projections indicated a 6.1% decline in the ASMR and a 1.8% decrease in the ASDR by 2036.

**Conclusion:**

The escalating burden of high FPG-driven CRC underscores its growing role in CRC epidemiology, particularly in aging populations and rapidly urbanizing regions. Persistent gender and socioeconomic disparities necessitate region-specific interventions that integrate diabetes management with CRC screening and equitable access to novel therapies. These findings advocate for prioritizing metabolic risk control in global CRC prevention frameworks to mitigate future burdens.

## Introduction

Colon and rectal cancer (CRC) is the third most prevalent tumor diagnosed and the second leading cause of cancer-related deaths, placing a heavy burden on the public health system (1). According to the latest Global Burden of Disease (GBD) data, there were 2.19 million new CRC cases, 1.04 million CRC deaths, and 24.4 million disability-adjusted life years (DALYs) lost in 2021 (2). Various pathways promote CRC (3), and both genetic and modifiable environmental risk factors contribute to CRC (4–6). While the epidemiological characteristics of CRC attributable to traditional risk factors have been extensively investigated (7–9), accumulating evidence shows that obesity, metabolic syndrome, dyslipidemia, and hypertension are also associated with an increased risk of CRC, most likely through common biological pathways. Against this background, the present study specifically focuses on high fasting plasma glucose (FPG) as a key, quantifiable metabolic risk factor, using the GBD 2021 framework to characterize the global, regional, and national burden of CRC attributable to high FPG and to place it in the context of sociodemographic development.

Hyperglycemia promotes CRC occurrence and development through multiple mechanisms. In addition, the global burden pattern of CRC is undergoing transformation due to urbanization, westernized diets, and population aging. In the past three decades, the global burden of diabetes has increased (10), contributing to the burden of CRC. Currently, there is still a gap in epidemiological research on high FPG-derived CRC, and cross-regional heterogeneity has not been systematically evaluated. In particular, the epidemiological pattern changes of CRC attributable to high FPG between high and low sociodemographic index (SDI) regions are still unclear. This gap directly hinders the scientific formulation of region-specific prevention and control policies and the effective implementation of evidence-based intervention measures.

Based on the GBD 2021 data, this study comprehensively analyzed the changes in the number of deaths and DALYs of CRC attributable to high FPG at the global, regional, and national levels. The risk heterogeneity of different SDI regions, ages, and sexes was further revealed. Furthermore, we revealed the current status of health inequality in high FPG-derived CRC based on the SDI and forecast the trend of the disease in the next 15 years.

## Methods

### Data source

All data used in this study were derived from the Global Burden of Disease (GBD) 2021 study, coordinated by the Institute for Health Metrics and Evaluation (IHME). The GBD 2021 database compiles standardized estimates of incidence, mortality, and disability for 371 diseases and injuries and 88 risk factors across 204 countries and territories from 1990 to 2021 (11). The underlying input data were systematically identified and extracted from multiple sources, including population-based surveys, disease registries, cancer registries, hospital and outpatient records, health insurance claims, vital registration and verbal autopsy systems, and other published and unpublished epidemiological studies. These data are catalogued in the Global Health Data Exchange (GHDx), which provides detailed metadata on the study design, population, and measurement methods. This study extracted data on deaths and DALYs of CRC attributable to high FPG in GBD 2021.

Definition of CRC (11): According to the International Classification of Diseases (ICD) codes mapped to non-fatal causes and injuries in the GBD 2021, the ICD-10 codes for colon and rectum cancer are C18 (malignant neoplasm of the colon, including the cecum, ascending, transverse, descending, and sigmoid colon), C19.0 (malignant neoplasm of the rectosigmoid junction), C20 (malignant neoplasm of the rectum), and C21–C21.8 (malignant neoplasms of the anus and anal canal), and the ICD-9 codes are 153–154.9 (malignant neoplasms of the colon, rectosigmoid junction, rectum, and anus), 209.1–209.17 (malignant carcinoid tumors of the colon, rectum, and anal canal), V10.05–V10.06 (personal history of malignant neoplasm of the large intestine and rectum), V76.41 (special screening for malignant neoplasm of the rectum), and V76.5–V76.52 (special screening for malignant neoplasm of the intestine, including the colon and rectum).

Definition of high FPG (12): High FPG is measured as the mean FPG in a population, where FPG is a continuous exposure in units of mmol/L. As FPG is along a continuum, we defined high FPG as any level above the theoretical minimum-risk exposure level, which is 4.9 mmol/L–5.3 mmol/L. The relative risks for high FPG as a risk factor for colorectal cancer were obtained from the GBD comparative risk assessment, which was derived from meta-analyses of prospective epidemiological studies and incorporated into the standard GBD risk–outcome modeling pipeline.

### Analytical framework and model

The GBD first applies systematic quality control and standardization to data from multiple sources (surveillance systems, registries, surveys, etc.), including harmonizing case and cause-of-death coding, checking internal consistency, and identifying and correcting implausible or contradictory records. Different data sources are then integrated within a unified modeling framework to reduce measurement errors and methodological heterogeneity. For country/region–age–sex–year strata with sparse or missing observations, GBD does not use simple pointwise imputation but instead relies on a series of hierarchical Bayesian models that “borrow strength” across time, space, and covariates. For example, incidence and prevalence are estimated using the DisMod-MR 2.1 Bayesian meta-regression platform, which pools all available data and uses region-level covariates to predict values in data-sparse settings, explicitly addressing issues such as missing data and inconsistency. Risk factor exposures are often modeled with spatiotemporal Gaussian process regression (ST-GPR), and many causes of death are estimated using the Cause of Death Ensemble model (CODEm). Thus, missing or sparse data are treated probabilistically within these models, with uncertainty reflected in 95% uncertainty intervals rather than through case deletion or single imputation.

### Projection using a Bayesian age–period–cohort model

To project the mortality and DALY rates of colorectal cancer attributable to high FPG from 2022 to 2036, we used a Bayesian age–period–cohort (BAPC) model implemented in the BAPC package in R. The model was fitted separately for each sex using annual data from 1990 to 2021. Age-, period-, and cohort-specific rates were modeled on the log scale and decomposed into an overall intercept and age, period, and cohort effects.

Following the default recommendations of the BAPC package, we assigned a vague normal prior with a large variance to the overall intercept and specified second-order random-walk priors for the age, period, and cohort effects to ensure smooth temporal patterns. The corresponding precision (smoothing) parameters were assigned weakly informative gamma priors. This specification allows borrowing of information across adjacent age groups, calendar years, and birth cohorts and has been widely used in age-period-cohort projections.

Posterior estimation was performed using the Markov chain Monte Carlo method provided by the BAPC package. We ran three parallel chains, each with 10,000 iterations, discarded the first 5,000 iterations as burn-in, and used a thinning interval of 10 to reduce autocorrelation, yielding 1,500 posterior samples for each of the parameters. Convergence was evaluated by visual inspection of the trace plots and by calculating Gelman–Rubin diagnostics; all R-hat values were <1.10, indicating satisfactory convergence. Based on the converged posterior samples, we obtained posterior predictive distributions for future calendar years (2022–2036) and summarized the projected age-standardized mortality and DALYs rates with their 95% uncertainty intervals.

### Statistical analysis

All rates were age-standardized using the GBD standard population, and 95% uncertainty intervals (UIs) for DALY and deaths were calculated by 1,000 bootstrap sampling, covering input data, model parameters, and residual variation.

In line with the GBD comparative risk assessment framework, we calculated the population attributable fraction (PAF) for CRC as the ratio of deaths or DALYs attributable to high FPG to total CRC deaths or DALYs in each region.

Joinpoint regression analysis: NCI Joinpoint 4.9.1.0 software was used to calculate the annual percentage change (APC) and average annual percentage change (AAPC) of the age-standardized rates from 1990 to 2021. The model determined the trend turning point through the Monte Carlo permutation test and selected the optimal number of segments (minimizing the Bayesian Information Criterion (BIC)). A positive AAPC indicates an increasing trend in burden over time, whereas a negative AAPC indicates a decreasing trend. Statistical significance was determined by whether the 95% confidence interval of the AAPC excluded 0. An AAPC of +1% implies that if the trend continues, the rate will increase at an average annual rate of 1% during the study period.

Health inequality analysis: The SDI quintile method was used to calculate the rate differences between the different SDI groups, and the slope index of inequality (SII) and concentration index (ConI) were used to quantify socioeconomic-related health inequalities. The SII measures absolute socioeconomic inequality, with larger absolute values indicating a wider burden gap between the highest and lowest SDI groups. The ConI is a measure of relative inequality, noting that negative values indicate that the disease burden is disproportionately concentrated in low-SDI (less advantaged) populations and that a decrease in the absolute value of ConI suggests that relative inequalities are narrowing.

## Results

### Global burden of high FPG-derived CRC

**Figure 1:**
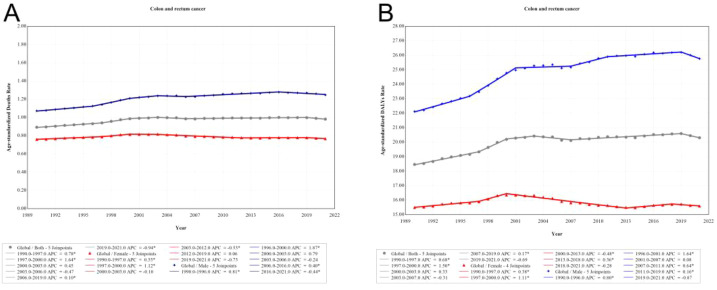
Joinpoint regression analysis of global deaths, and DALYs of colon and rectum cancer attributable to high fasting plasma glucose from 1990 to 2021. **(A)** Deaths, **(B)** DALYs.

The number of CRC deaths attributable to high FPG increased from 31,907 (95% UI: 16,053 to 48,058) in 1990 to 82,421 (95% UI: 42,427–125,402) in 2021, and DALYs loss increased from 715,716 (95% UI: 358,249–1,089,712) in 1990 to 1,750,923 (95% UI: 900,573 to 2,657,995) in 2021. In addition, in 2021, the global age-standardized mortality rate (ASMR) of CRC attributable to high FPG was 0.98 (95% UI: 0.51 to 1.49) per 100,000 person-years, and the age-standardized DALYs rate (ASDR) was 20.31 (95% UI: 10.46 to 30.81) per 100,000 person-years, both of which experienced an average annual increase of 0.31% between 1990 and 2021 (**Table 1**). Joinpoint analysis showed that the global ASMR and ASDR showed slow growth in 2006–2019 (APC = 0.10%) and 2007–2019 (APC = 0.17%), respectively, and then showed a downward trend in 2019–2021 (ASMR APC = −0.94%, ASDR APC = −0.69%) (**Figure 1**).

**Table 1:** Deaths and DALYs of colon and rectum cancer attributable to high fasting plasma glucose and their average annual percent changes (AAPCs) from 1990 to 2021 globally, across different SDI regions, and in 21 regions.

Location	Deaths (95%UI)					DALYs (95%UI)				
	Number		ASR per 100,000			Number		ASR per 100,000		
	1990	2021	1990	2021	Average annual percent change (95% CI)	1990	2021	1990	2021	Average Annual Percent Change (95% CI)
Global	31,907 (16,053, 48,058)	82,421 (42,427, 125,402)	0.89 (0.45, 1.34)	0.98 (0.51, 1.49)	0.31 (0.20, 0.43)	715,716 (358,249, 1,089,712)	1,750,923 (900,573, 2,657,995)	18.46 (9.27, 28.03)	20.31 (10.46, 30.81)	0.31 (0.19, 0.43)
High SDI	15,050 (7,739, 22,450)	30,579 (15,404, 46,082)	1.33 (0.69, 1.99)	1.33 (0.67, 1.99)	−0.01 (−0.07, 0.06)	308,579 (156,944, 463,035)	577,527 (295,230, 861,475)	27.71 (14.1, 41.57)	27.65 (14.2, 41.32)	−0.03 (−0.14, 0.09)
High-middle SDI	9,033 (4,496, 13,805)	23,255 (12,027, 35,689)	0.97 (0.49, 1.48)	1.17 (0.61, 1.8)	0.65 (0.46, 0.84)	207,544 (103,765, 318,802)	494,756 (252,815, 766,069)	20.94 (10.47, 32.11)	24.87 (12.71, 38.46)	0.57 (0.38, 0.76)
Low SDI	657 (320, 1,024)	1,889 (911, 2,924)	0.34 (0.16, 0.53)	0.44 (0.22, 0.69)	0.92 (0.78, 1.05)	16,746 (8,238, 26,052)	45,699 (22,154, 70,621)	7.46 (3.65, 11.66)	9.27 (4.47, 14.31)	0.72 (0.61, 0.83)
Low-middle SDI	1,638 (827, 2,508)	6,938 (3,441, 10,555)	0.3 (0.15, 0.46)	0.52 (0.26, 0.79)	1.82 (1.71, 1.93)	42,473 (21,671, 65,607)	170,488 (84,721, 258,664)	6.86 (3.48, 10.53)	11.71 (5.82, 17.78)	1.77 (1.70, 1.83)
Middle SDI	5,479 (2,666, 8,462)	19,635 (10,122, 30,542)	0.61 (0.29, 0.93)	0.77 (0.4, 1.2)	0.84 (0.61, 1.07)	139,272 (68,101, 216,722)	459,950 (235,527, 714,076)	13.39 (6.53, 20.72)	16.92 (8.68, 26.32)	0.81 (0.58, 1.03)
Region										
Andean Latin America	73 (37, 114)	435 (215, 690)	0.39 (0.2, 0.61)	0.77 (0.38, 1.21)	2.16 (1.74, 2.57)	1,571 (793, 2,487)	8,971 (4,378, 14,236)	7.96 (4.03, 12.55)	15.38 (7.52, 24.39)	2.12 (1.69, 2.56)
Australasia	334 (166, 502)	681 (354, 991)	1.42 (0.71, 2.13)	1.18 (0.61, 1.72)	−0.63 (−0.79, −0.46)	7,249 (3,632, 10,946)	13,217 (6,762, 19,457)	30.84 (15.43, 46.5)	24.9 (12.66, 37.04)	−0.74 (−0.89, −0.58)
Caribbean	260 (129, 395)	746 (370, 1,172)	1.06 (0.53, 1.61)	1.38 (0.68, 2.17)	0.90 (0.80, 1.01)	5,460 (2,747, 8,330)	15,517 (7,784, 24,658)	21.32 (10.74, 32.48)	28.77 (14.44, 45.74)	1.00 (0.85, 1.16)
Central Asia	165 (81, 251)	403 (196, 623)	0.37 (0.18, 0.55)	0.54 (0.26, 0.83)	1.35 (0.94, 1.77)	4,239 (2,078, 6,448)	9,936 (4,836, 15,266)	8.87 (4.36, 13.53)	12.06 (5.85, 18.57)	1.08 (0.58, 1.57)
Central Europe	2,067 (1,052, 3,061)	5,117 (2,627, 7,697)	1.42 (0.72, 2.1)	2.16 (1.11, 3.25)	1.38 (1.30, 1.46)	44,997 (22,687, 66,970)	101,124 (51,636,151,350)	29.72 (15.04, 44.26)	44.68 (22.83, 66.76)	1.33 (1.26, 1.41)
Central Latin America	418 (209, 631)	2,117 (1,084, 3,247)	0.57 (0.28, 0.85)	0.87 (0.44, 1.34)	1.39 (1.22, 1.56)	9,494 (4,767, 142,11)	49,644 (25,722, 75,378)	11.66 (5.84, 17.51)	19.66 (10.17, 29.88)	1.70 (1.59, 1.81)
Central Sub-Saharan Africa	88 (45, 140)	263 (126, 430)	0.48 (0.24, 0.75)	0.58 (0.28, 0.96)	0.62 (0.55, 0.69)	2,336 (1,182, 3,704)	6,947 (3,329, 11,311)	10.55 (5.33, 16.74)	12.72 (6.1, 20.82)	0.61 (0.53, 0.69)
East Asia	6,527 (3,219, 9,997)	19,468 (9,807, 30,265)	0.87 (0.43, 1.33)	0.92 (0.47, 1.44)	0.30 (0.00, 0.59)	169,695 (82,740, 262,486)	451,052 (222,989, 702,618)	19.33 (9.54, 29.8)	20.56 (10.15, 31.98)	0.27 (−0.04, 0.59)
Eastern Europe	1,888 (947, 2,885)	3,869 (1,974, 5,849)	0.68 (0.34, 1.04)	1.06 (0.54, 1.61)	1.45 (0.88, 2.02)	44,802 (22,524, 68,640)	82,734 (42,262, 124,535)	15.72 (7.92, 24.13)	23.09 (11.8, 34.79)	1.34 (0.72, 1.97)
Eastern Sub-Saharan Africa	251 (119, 405)	703 (337, 1,109)	0.41 (0.2, 0.67)	0.54 (0.26, 0.85)	0.90 (0.80, 0.99)	6,077 (,2910, 9,696)	15,724 (7,534, 24,539)	8.59 (4.1, 13.81)	10.34 (4.98, 16.3)	0.60 (0.52, 0.68)
High-income Asia Pacific	2,674 (1,384, 3,951)	7,308 (3,687, 10,994)	1.4 (0.73, 2.06)	1.29 (0.65, 1.93)	−0.32 (−0.62, −0.01)	58,362 (30,330, 85,989)	122,009 (62,513, 182,120)	29.07 (15.14, 42.8)	25.57 (12.97, 38.3)	−0.46 (−0.72, −0.19)
High-income North America	5,105 (2,653, 7,625)	9,626 (4,911, 14,504)	1.4 (0.73, 2.09)	1.41 (0.72, 2.12)	−0.01 (−0.17, 0.16)	106,584 (54,673, 157,151)	200,781 (103,845, 300,344)	30.31 (15.58, 44.69)	31.31 (16.16, 46.55)	0.11 (−0.20, 0.42)
North Africa and Middle East	770 (392, 1,186)	3,626 (1,776, 5,587)	0.53 (0.27, 0.82)	0.91 (0.45, 1.41)	1.78 (1.70, 1.86)	19,080 (9,610, 29,426)	87,065 (42,713, 133,798)	11.45 (5.82, 17.66)	19.34 (9.52, 29.77)	1.71 (1.64, 1.78)
Oceania	13 (7, 20)	37 (19, 58)	0.55 (0.29, 0.86)	0.59 (0.3, 0.94)	0.23 (0.12, 0.34)	348 (181, 552)	979 (506, 1,542)	11.94 (6.22, 18.73)	13 (6.65, 20.41)	0.27 (0.14, 0.40)
South Asia	1,355 (648, 2,067)	5,426 (2,718, 8,182)	0.26 (0.12, 0.4)	0.39 (0.19, 0.59)	1.41 (1.12, 1.71)	36,978 (17,750, 56,172)	137,712 (69,392, 207,550)	6.16 (2.95, 9.35)	9.07 (4.57, 13.65)	1.29 (1.15, 1.43)
Southeast Asia	1,110 (542, 1,735)	5,368 (2,706, 8,656)	0.51 (0.25, 0.81)	0.92 (0.46, 1.49)	1.92 (1.77, 2.07)	26,680 (13,133, 41,547)	123,840 (62,644, 198,168)	10.78 (5.28, 16.85)	19.16 (9.69, 30.7)	1.89 (1.70, 2.08)
Southern Latin America	492 (245, 752)	1,402 (711, 2,172)	1.1 (0.55, 1.68)	1.56 (0.79, 2.41)	1.16 (0.87, 1.46)	10,310 (5,193, 15,726)	27,930 (14,222, 43,037)	22.27 (11.19, 34.04)	31.83 (16.18, 48.88)	1.19 (0.92, 1.46)
Southern Sub-Saharan Africa	107 (53, 167)	428 (203, 663)	0.47 (0.23, 0.73)	0.86 (0.4, 1.33)	1.95 (1.62, 2.27)	2,274 (1,126, 3,521)	9,786 (4,681, 15,062)	8.93 (4.41, 13.82)	17.44 (8.34, 26.87)	2.18 (1.82, 2.54)
Tropical Latin America	494 (245, 755)	2,390 (1,229, 3,546)	0.61 (0.3, 0.94)	0.95 (0.49, 1.4)	1.45 (1.28, 1.61)	11,465 (5,702, 17,469)	55,135 (28,144, 82,455)	12.78 (6.36, 19.58)	21.25 (10.86, 31.78)	1.65 (1.47, 1.83)
Western Europe	7,550 (3,791, 11,405)	12,373 (6,101, 19,098)	1.23 (0.62, 1.86)	1.15 (0.57, 1.77)	−0.22 (−0.32, −0.12)	143,915 (72,575, 216,942)	216,393 (106,980, 331,728)	24.36 (12.34, 36.76)	22.7 (11.26, 34.71)	−0.25 (−0.38, −0.11)
Western Sub-Saharan Africa	168 (83, 264)	635 (294, 1,002)	0.23 (0.11, 0.36)	0.4 (0.19, 0.64)	1.86 (1.79, 1.93)	3,797 (1,883, 5,978)	14,428 (6,672, 22,827)	4.56 (2.27, 7.18)	7.92 (3.67, 12.51)	1.79 (1.68, 1.90)

SDI, socio-demographic index; AAPC, average annual percent change; DALYs, disability-adjusted life years; ASR, age-standardized rate; UI, uncertainty interval; CI, confidence interval.

### Regional burden of high FPG-derived CRC

For the GBD 21 regions, East Asia had the highest number of deaths (19,468, 95% UI: 9,807–30,265) and DALYs loss (45,1052, 95% UI: 222,989–702,618) due to CRC attributable to high FPG, whereas Central Europe had the highest ASMR (2.16, 95% CI: 1.11–3.25 per 100,000 person-years) and ASDR (44.68, 95% CI: 22.83–66.76 per 100,000 person-years). From 1990 to 2021, most regions showed a rapid growth trend in the ASMR and ASDR, with only Australia, high-income Asia Pacific, and Western Europe showing a downward trend (**Table 1**). Globally, the PAF of CRC deaths attributable to high FPG increased from 14.29% in 1990 to 16.93% in 2021, with the highest values observed in Central Latin America (up to 21.51%). The PAF of CRC DALYs attributable to high FPG increased from 13.32% in 1990 to 15.69% in 2021, with the highest values observed in Central Latin America (up to 20.37%). From 1990 to 2021, the PAF increased in all regions (**Supplementary Table 1**).

### National burden of high FPG-derived CRC

**Figure 2:**
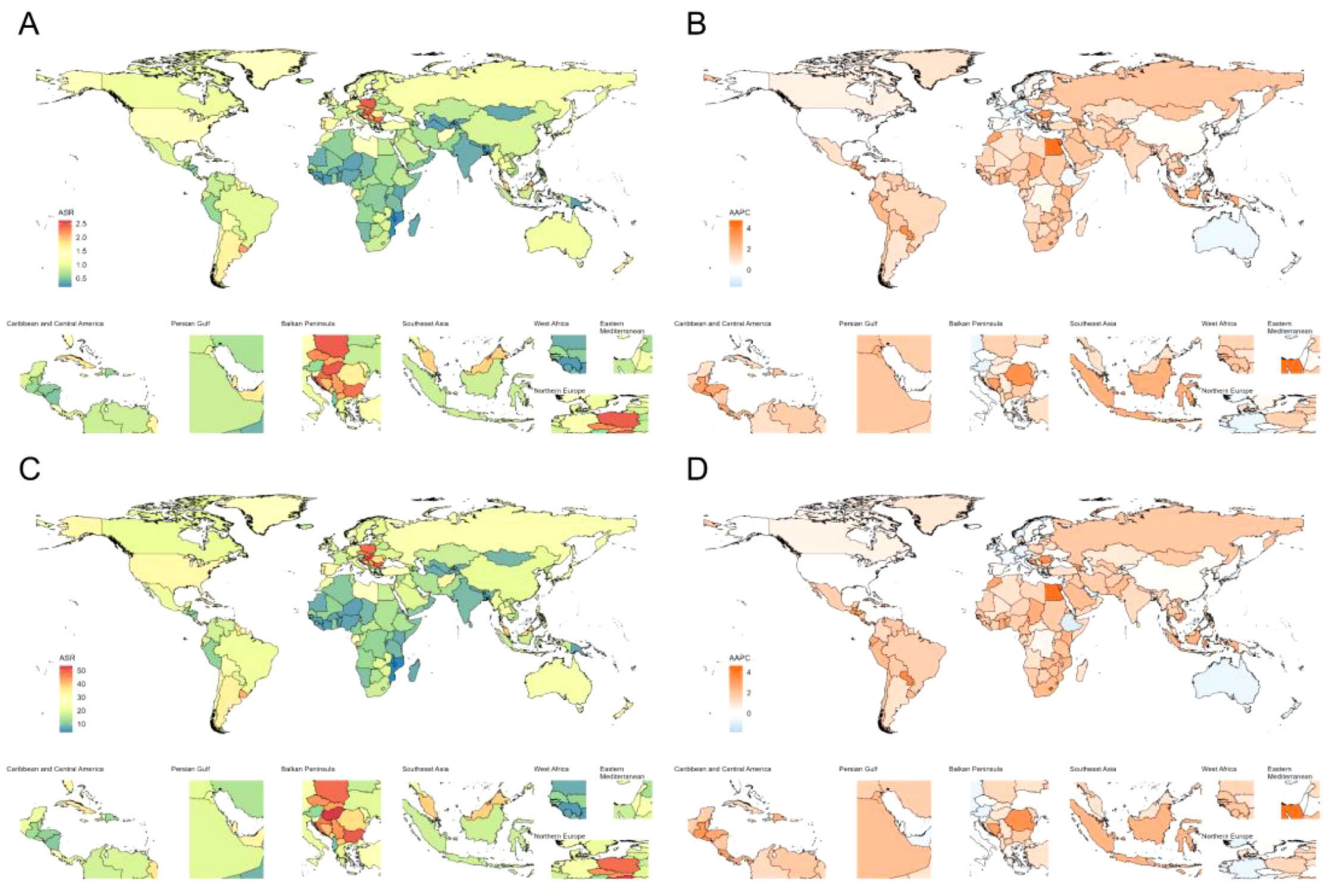
Age-standardized rates of deaths, and DALYs, along with their average annual percent change (AAPC) from 1990 to 2021, in colon and rectum cancer attributable to high fasting plasma glucose across countries. **(A)** Age-standardized mortality rates; **(B)** AAPC of age-standardized mortality rates; **(C)** Age-standardized DALYs rates; **(D)** AAPC of age-standardized DALYs rates. DALYs, disability-adjusted life years.

At the national level, China (18,440, 95% UI: 9,208–28,705), Japan (6,208, 95% UI: 3,124–9,300), and the United States (8,793, 95% UI: 4,511–13,193) had the highest number of deaths due to CRC attributable to high FPG, whereas China (429,386, 95% UI: 210,526–671,812), the United States (185,505, 95% UI: 95,872–276,078), and India (110,349, 95% UI: 55,527–169,069) had the highest number of DALYs lost. Furthermore, the country with the highest ASMR was Barbados (2.62, 95% UI: 1.35–4.21 per 100,000 person-years), while the country with the highest ASDR was Hungary (53.69, 95% CI: 26.01–85.07 per 100,000 person-years) (**Figures 2A, C**). Trend analysis showed that between 1990 and 2021, Cape Verde, Egypt, and Lesotho experienced rapid growth in ASMR and ASDR (**Figures 2B, D**).

### Age and gender differences in high FPG-derived CRC burden

**Figure 3:**
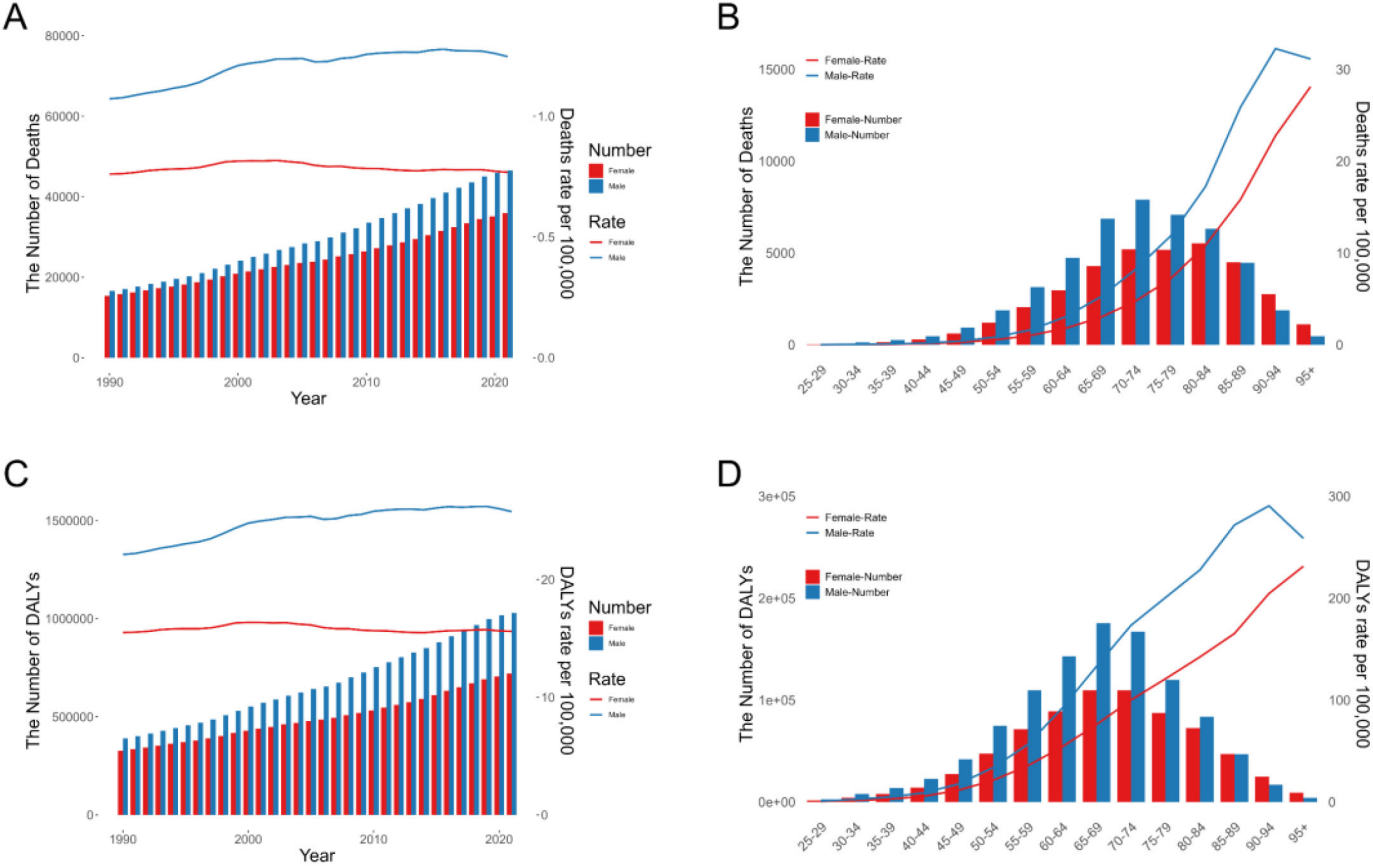
The global burden of colon and rectum cancer attributable to high fasting plasma glucose by year, by gender, and by age group. **(A)** The number and age-standardized rate of deaths from the global burden by year and sex. **(B)** The number and age-standardized rate of deaths from the global burden by age group and sex in 2021. **(C)** The number and age-standardized rate of DALYs from the global burden by year and sex. **(D)** The number and age-standardized rate of DALYs from the global burden by age group and sex in 2021. DALYs, disability-adjusted life years.

**Figure 4:**
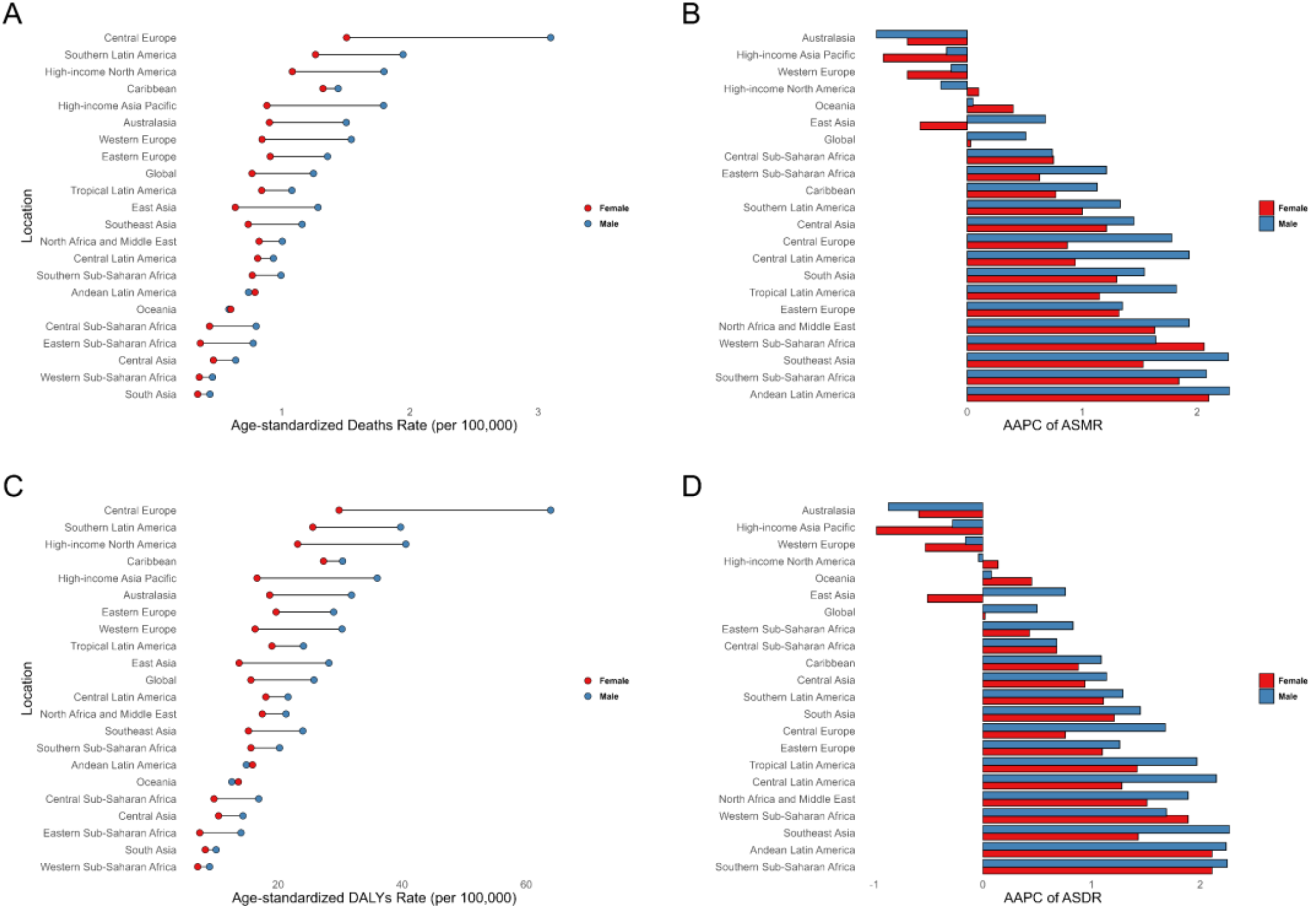
Age-standardized rates of deaths, and DALYs, along with their average annual percent change from 1990 to 2021, in colon and rectum cancer attributable to high fasting plasma glucose across 21 regions. **(A)** and **(B)** Deaths. **(C)** and **(D)** DALYs. DALYs, disability-adjusted life years.

When age was stratified by 5 years, the results showed that the peak of CRC deaths (13,108, 95% UI: 6,554–20,243) attributable to high FPG occurred in those aged 70–74 years, while the peak of DALYs loss (284,822, 95% UI: 143,760–431,538) occurred in those aged 65–69 years. Both mortality and DALYs rates increased with age (**Table 2**, **Figure 3**). During 1990 and 2021, most age groups showed an increasing trend in mortality and DALYs rates, with the fastest growth in the 95+ population (AAPC for mortality rate = 1.28%, 95% CI: 1.15%–1.40%; AAPC for DALYs rate = 1.22%, 95% CI: 1.08%–1.36%) (**Table 2**). For the GBD 21 regions, the burden on males was significantly higher than that on females in most regions, and males showed a faster growth trend between 1990 and 2021 (**Figure 4**).

**Table 2:** Deaths and DALYs of colon and rectum cancer attributable to high fasting plasma glucose and their average annual percent changes (AAPCs) from 1990 to 2021 globally, across different age groups.

Age group	Deaths (95%UI)					DALYs (95%UI)				
	Number		ASR per 100,000			Number		ASR per 100,000		
	1990	2021	1990	2021	Average annual percent change (95% CI)	1990	2021	1990	2021	Average annual percent change (95% CI)
25–29 years	49 (23, 84)	65 (31, 102)	0.01 (0.01, 0.02)	0.01 (0.01, 0.02)	−0.04 (−0.32, 0.23)	3,151 (1,479, 5,339)	4,178 (2,020, 6,595)	0.71 (0.33, 1.21)	0.71 (0.34, 1.12)	−0.01 (−0.29, 0.26)
30–34 years	106 (47, 168)	199 (98, 318)	0.03 (0.01, 0.04)	0.03 (0.02, 0.05)	0.64 (0.10, 1.19)	6,202 (2,778, 9,820)	11,798 (5,822, 18,991)	1.61 (0.72, 2.55)	1.95 (0.96, 3.14)	0.68 (0.16, 1.20)
35–39 years	228 (106, 383)	394 (193, 617)	0.06 (0.03, 0.11)	0.07 (0.03, 0.11)	0.29 (−0.00, 0.60)	12,244 (5,710, 20,519)	21,458 (10,479, 33,711)	3.48 (1.62, 5.83)	3.83 (1.87, 6.01)	0.34 (0.04, 0.64)
40–44 years	379 (187, 590)	739 (364, 1,119)	0.13 (0.07, 0.21)	0.15 (0.07, 0.22)	0.34 (0.20, 0.49)	18,526 (9,161, 28,722)	36,561 (17,946, 55,409)	6.47 (3.2, 10.03)	7.31 (3.59, 11.08)	0.38 (0.24, 0.52)
45–49 years	678 (333, 1043)	1,557 (797, 2,357)	0.29 (0.14, 0.45)	0.33 (0.17, 0.5)	0.42 (0.21, 0.64)	29,773 (14,660, 45,926)	69,183 (35,427, 104,601)	12.82 (6.31, 19.78)	14.61 (7.48, 22.09)	0.46 (0.25, 0.67)
50–54 years	1,367 (666, 2,091)	3,078 (1,559, 4,658)	0.64 (0.31, 0.98)	0.69 (0.35, 1.05)	0.24 (0.10, 0.38)	53,426 (26,002, 81,894)	122,071 (61,845, 186,200)	25.13 (12.23, 38.53)	27.44 (13.9, 41.85)	0.29 (0.15, 0.42)
55–59 years	2,313 (1,162, 3,589)	5,174 (2,664, 7,836)	1.25 (0.63, 1.94)	1.31 (0.67, 1.98)	0.16 (−0.05, 0.37)	79,538 (39,883, 123,327)	180,745 (92,652, 274,513)	42.95 (21.54, 66.59)	45.67 (23.41, 69.37)	0.20 (0.01, 0.40)
60–64 years	3,608 (1,812, 5,546)	7,688 (3,967, 11,686)	2.25 (1.13, 3.45)	2.4 (1.24, 3.65)	0.22 (−0.02, 0.46)	107,307 (53,930, 164,832)	231,955 (119,526, 352,953)	66.81 (33.58, 102.63)	72.48 (37.35, 110.28)	0.27 (0.02, 0.52)
65–69 years	4,679 (2,329, 7,007)	11,156 (5,663, 168,42)	3.79 (1.88, 5.67)	4.04 (2.05, 6.11)	0.23 (0.09, 0.37)	117,726 (58,651,176,121)	284,822 (143,760, 431,538)	95.24 (47.45, 142.48)	103.26 (52.12, 156.44)	0.27 (0.14, 0.40)
70–74 years	4,883 (2,418, 7,372)	13,108 (6,554, 20,243)	5.77 (2.86, 8.71)	6.37 (3.18, 9.83)	0.31 (0.07, 0.56)	101,408 (50,295,154,073)	276,591 (139,147, 425,016)	119.78 (59.41, 181.99)	134.37 (67.6, 206.48)	0.38 (0.22, 0.55)
75–79 years	5,585 (2,761, 8,326)	12,253 (6,346, 18,764)	9.07 (4.49, 13.53)	9.29 (4.81, 14.23)	0.10 (−0.04, 0.23)	92,809 (46,329, 138,551)	207,055 (106,900, 315,773)	150.77 (75.26, 225.08)	157 (81.06, 239.43)	0.15 (0.01, 0.30)
80–84 years	4,456 (2,276, 6,792)	11,839 (6,073, 18,257)	12.6 (6.43, 19.2)	13.52 (6.93, 20.85)	0.25 (0.12, 0.38)	58,124 (29,783, 88,590)	155,956 (80,338, 240,446)	164.3 (84.19, 250.43)	178.07 (91.73, 274.54)	0.28 (0.16, 0.40)
85–89 years	2,502 (1,272, 3,839)	8,970 (4,344, 13,877)	16.55 (8.42, 25.4)	19.62 (9.5, 30.35)	0.58 (0.39, 0.77)	25,980 (13,279, 39,723)	93,930 (45,818, 145,932)	171.93 (87.88, 262.87)	205.44 (100.21, 319.17)	0.60 (0.42, 0.79)
90–94 years	877 (447, 1,330)	4,626 (2,272, 7,056)	20.45 (10.43, 31.03)	25.86 (12.7, 39.44)	0.78 (0.65, 0.92)	7,839 (4,030, 11,894)	41,600 (20,580, 63,694)	182.93 (94.05, 277.57)	232.54 (115.04, 356.04)	0.80 (0.67, 0.94)
95+ years	198 (98, 305)	1,577 (755, 2,423)	19.47 (9.65, 30)	28.94 (13.85, 44.45)	1.28 (1.15, 1.40)	1,663 (825, 2,557)	13,020 (6,232, 20,027)	163.34 (81, 251.11)	238.88 (114.34, 367.44)	1.22 (1.08, 1.36)

SDI, Socio-Demographic Index; AAPC, Average Annual Percent Change; DALYs, Disability-Adjusted Life Years; ASR, Age-Standardized Rate; UI, uncertainty interval; CI, confidence interval.

### Association between high FPG-derived CRC burden and SDI

**Figure 5:**
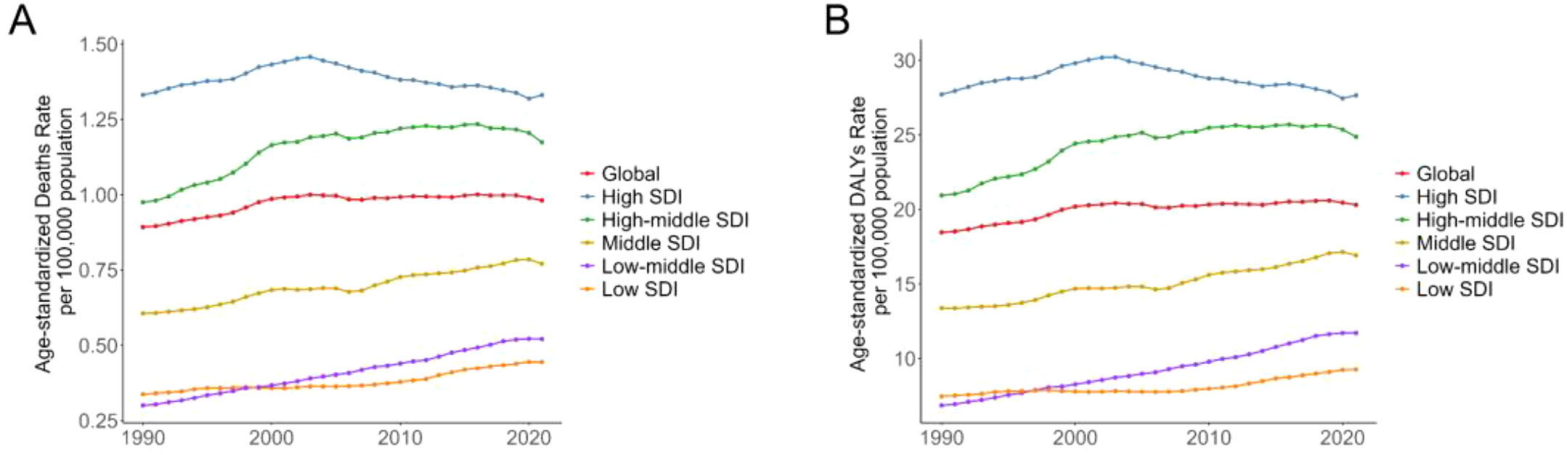
Age-standardized rates of deaths, and DALYs due to colon and rectum cancer attributable to high fasting plasma glucose, by SDI quintiles for both sexes, 1990–2021. **(A)** Deaths. **(B)** DALYs. DALYs, disability-adjusted life years; SDI, socio-demographic index.

At the regional level, the CRC burden attributable to high FPG levels was positively associated with SDI. High SDI regions had the most deaths (30,579, 95% UI: 15,404–46,082) and DALYs loss (577,527, 95% UI: 295,230–861,475), as well as the highest ASMR (1.33, 95% UI: 0.67–1.99 per 100,000 person-years) and ASDR (27.65, 95% UI: 14.2–41.32 per 100,000 person-years), while low SDI regions had the lowest burden (ASMR, 0.44 per 100,000 person-years, 95% UI: 0.22–0.69; ASDR, 9.27 per 100,000 person-years, 95% UI: 4.47–14.31). Trend analysis showed that except for the high SDI regions, which showed relatively stable ASMR and ASDR, other regions showed significantly rising ASMR and ASDR during 1990 and 2021, with the most significant increase in low-middle SDI regions (AAPC for ASMR = 1.82%, 95% CI: 1.71%–1.93%; AAPC for ASDR = 1.77%, 95% CI: 1.70%–1.83%) (**Table 1**, **Figure 5**).

**Figure 6:**
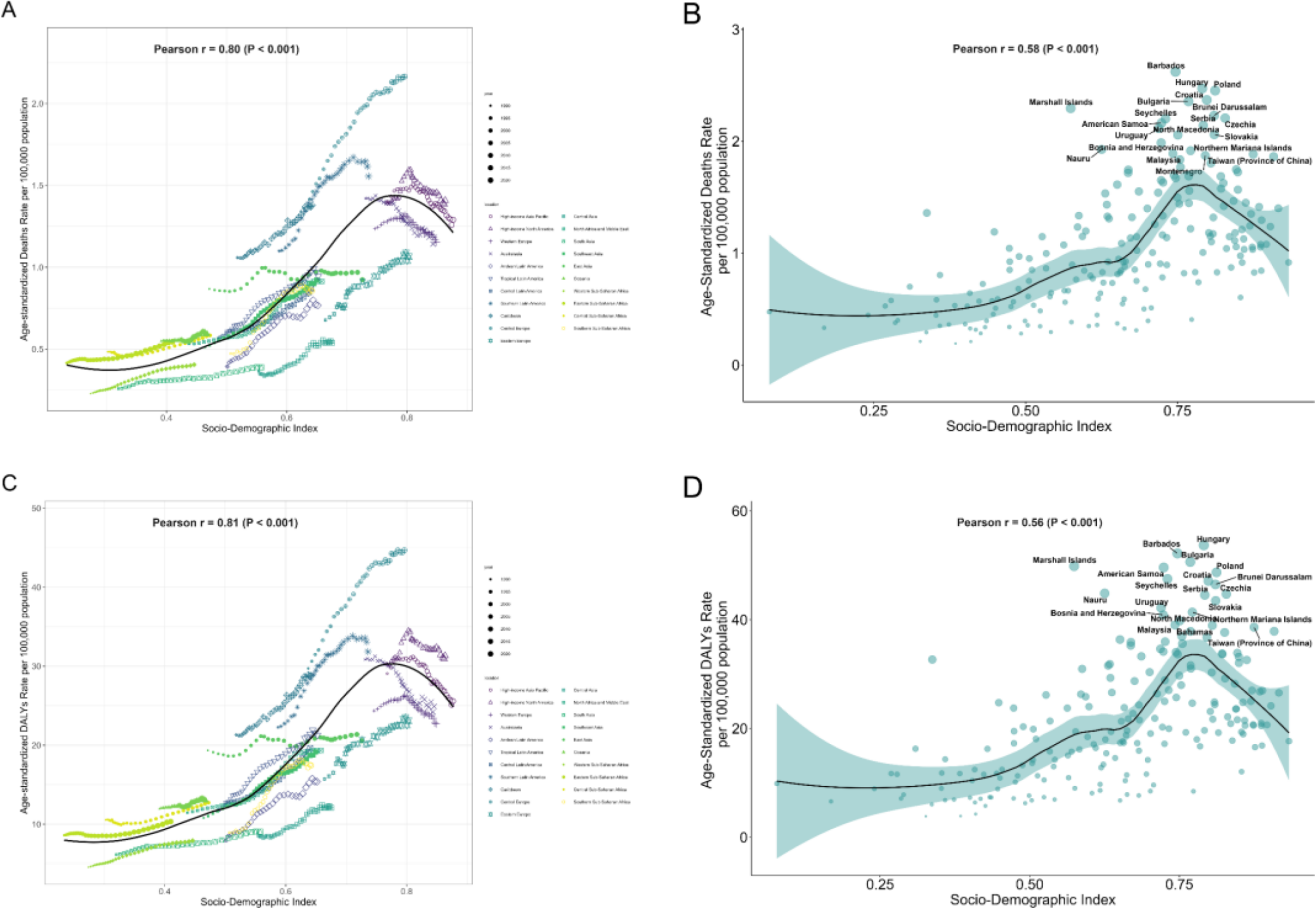
Burden of colon and rectum cancer attributable to high fasting plasma glucose across 21 regions and 204 countries by SDI. **(A)** ASMR for 21 regions by SDI from 1990 to 2021. **(B)** ASMR for 204 countries by SDI from 1990 to 2021. **(C)** ASDR for 21 regions by SDI from 1990 to 2021. **(D)** ASDR for 204 countries by SDI from 1990 to 2021. ASMR, age-standardized mortality rate; ASDR, age-standardized disability-adjusted life year rate; SDI, socio-demographic index.

Stratifying 21 GBD regions and 204 countries by SDI, we found that the ASMR and ASDR of CRC attributable to high FPG were significantly and positively correlated with SDI. In the 21 GBD regions, the correlation coefficient between the ASMR and SDI was 0.80 (p <0.001), and the correlation coefficient between the ASDR and SDI was 0.81 (p < 0.001) (**Figures 6A, B**). In 204 countries or territories, the correlation coefficient between the ASMR and SDI was 0.58 (p <0.001), and the correlation coefficient between the ASDR and SDI was 0.56 (p <0.001) (**Figures 6C, D**).

### Health inequality analysis

**Figure 7:**
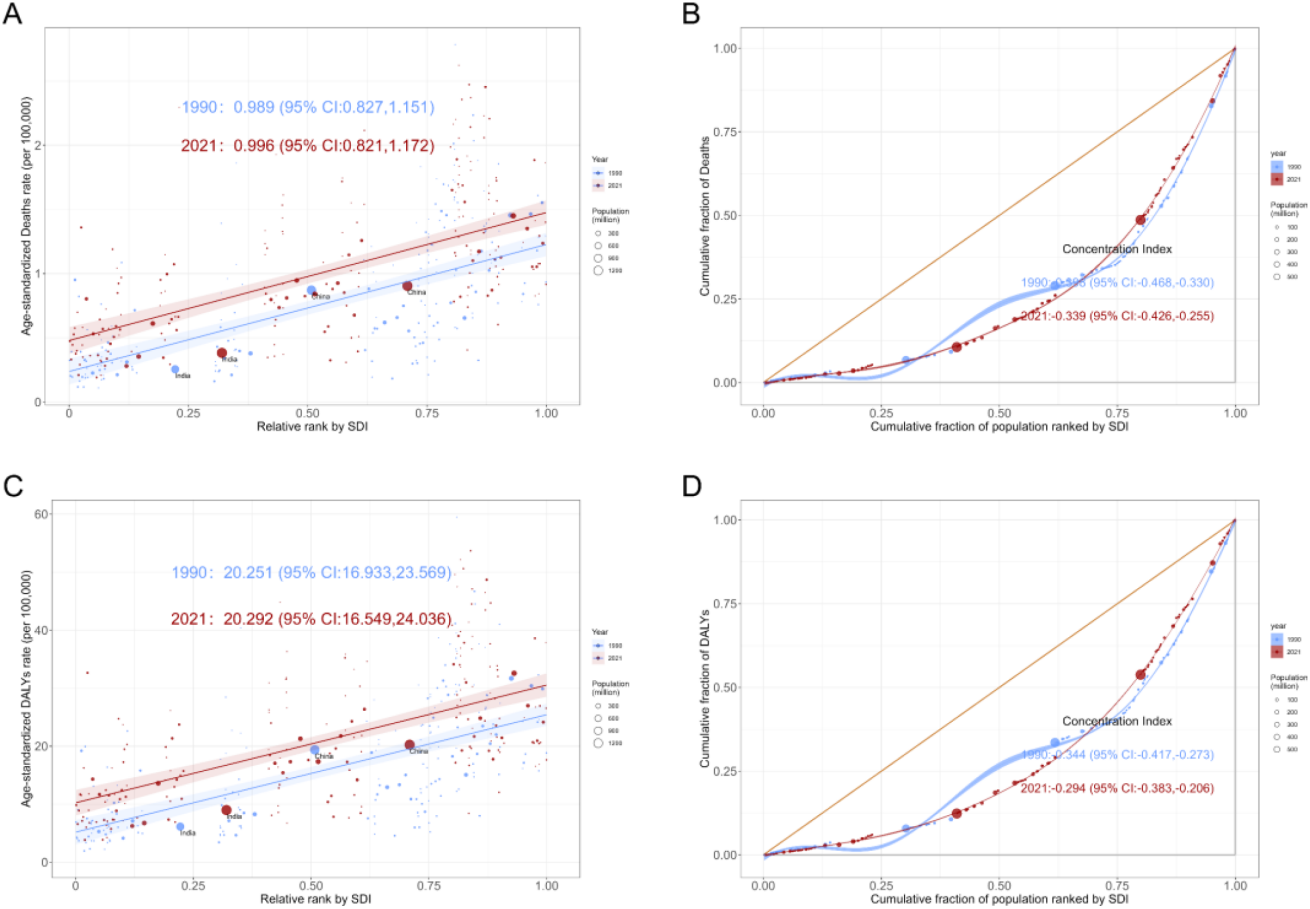
Slope index of inequality (left) and concentration index (right) curves for colon and rectum cancer attributable to high fasting plasma glucose in 1990 and 2021. **(A)** Deaths. **(B)** DALYs. DALYs, disability-adjusted life years.

The results of the health inequality analysis showed that the SII of ASMR increased from 0.989 (95% CI: 0.827–1.151) in 1990 to 0.996 (95% CI: 0.821–1.172) in 2021, while the SII of ASDR increased from 20.251 (95% CI: 16.933–23.569) in 1990 to 20.292 (95% CI: 16.949–24.036) in 2021, revealing a slight increase in absolute inequality. In contrast, the ConI of the ASMR increased from −0.398 (95% CI: −0.468 to −0.330) in 1990 to −0.339 (95% CI: −0.426 to −0.255) in 2021, while the ConI of the ASDR increased from −0.334 (95% CI: −0.417 to −0.273) in 1990 to −0.294 (95% CI: −0.383 to −0.206) in 2021, revealing that relative inequality was narrowing (**Supplementary Table 2**, **Figure 7**).

### Forecasting the future trend of high FPG-derived CRC

**Figure 8:**
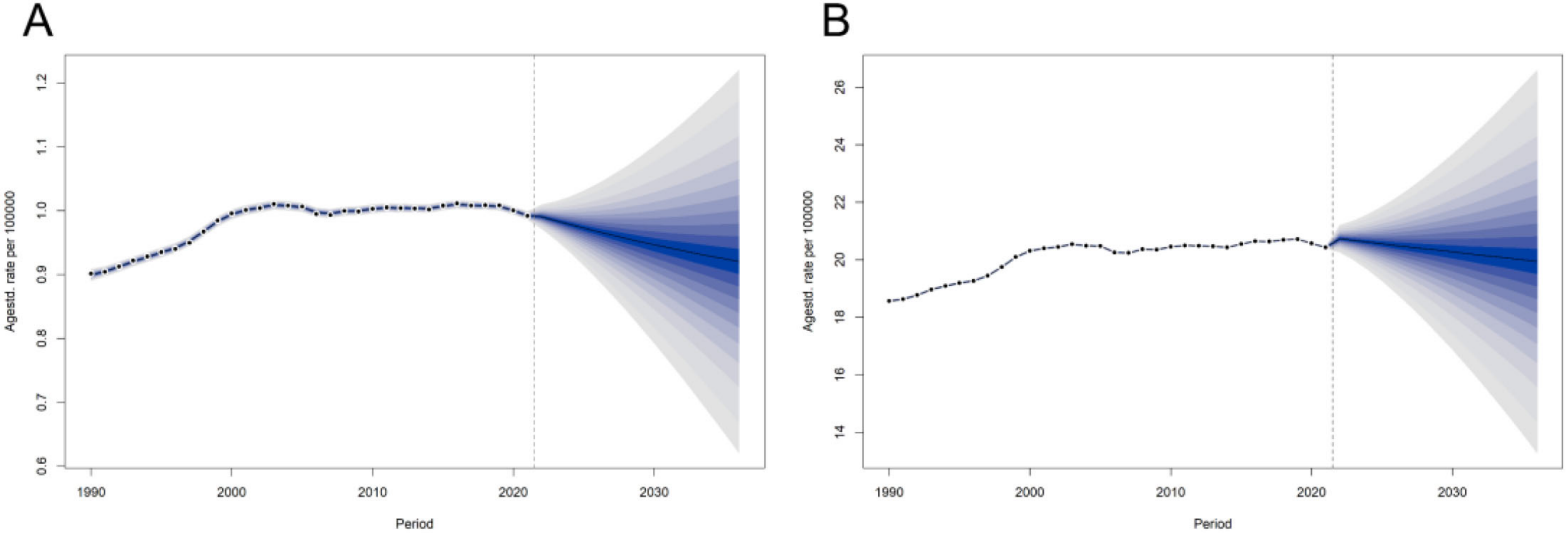
Trends and forecasts of the burden of colon and rectum cancer attributable to high fasting plasma glucose globally until 2036. **(A)** Deaths. **(B)** DALYs, disability-adjusted life years.

The BAPC model predicted that by 2036, the number of deaths caused by CRC attributable to high FPG would increase to 82,622 (95% UI: 55,764–109,479), and the DALYs loss would increase to 1,790,058 (95% UI: 1,193,335–2,386,781). In contrast, the ASMR would drop to 0.92 (95% UI: 0.62–1.22) and ASDR would drop to 19.94 (95% UI: 13.29–26.59) by 2036 (**Supplementary Table 3**, **Figure 8**).

## Discussion

This study revealed the evolution characteristics of the global disease burden of CRC related to high FPG based on the GBD 2021 data. We found that the age-standardized mortality and DALYs rates of CRC attributable to high FPG continued to increase during the study period. The marked increase in the absolute number of deaths and DALYs may primarily reflect population growth and aging, whereas the slight decline in ASMR is likely attributable to improvements in prevention, early detection, and treatment. An increase in the ASDR indicates that the impact of colorectal cancer attributable to high FPG on healthy life expectancy is intensifying, with more years of healthy life lost per capita. Notably, mortality and DALY rates in the male group were higher than those in women. Although the disease burden is currently heavy in regions with high SDI, the trend from 1990 to 2021 was relatively stable, whereas the mortality and DALY rates in low SDI regions are still increasing rapidly. Predictions based on the BAPC model showed that by 2036, the global mortality and DALY rates of CRC attributable to high FPG may decrease by 6.1% and 1.8%, respectively. The decline in age-standardized rates reflects potential improvements in risk factor control, screening, and care among individuals of a given age, whereas population growth and aging can still lead to an increase in the absolute number of events. The projected values and their 95% UIs should be interpreted as probabilistic forecasts under the assumption that past trends and current intervention patterns will persist.

From a biological perspective, several interconnected pathways may explain how high FPG promotes CRC occurrence, progression, and metastasis. First, high FPG provides tumor and premalignant cells with an abundant carbon source, facilitating energy metabolism reprogramming and the Warburg effect (13, 14). Enhanced glycolysis, together with increased flux through the pentose phosphate and lipid synthesis pathways, supplies ATP, nucleotides, and membrane lipids that support the rapid clonal expansion of dysplastic colonic epithelium. Second, hyperglycemia is frequently accompanied by hyperinsulinemia and activation of the insulin/IGF-1 axis (15). Binding of insulin and IGF-1 to their receptors on epithelial and stromal cells activates the PI3K/AKT/mTOR and RAS/RAF/MEK/ERK signaling pathways, which promotes cell cycle progression, inhibits apoptosis, and enhances protein synthesis, thereby facilitating the adenoma-carcinoma sequence and resistance to cell death (16, 17). Third, sustained high glucose levels induce excessive production of reactive oxygen species and advanced glycation end products, leading to DNA damage, genomic instability, and activation of NF-κB–driven inflammatory pathways (18, 19). The resulting chronic inflammatory microenvironment, characterized by increased cytokine levels and COX-2–derived prostaglandins, further supports angiogenesis, immune evasion, and invasive growth. Fourth, Westernized, high-sugar, and high-fat diets that commonly accompany hyperglycemia can drive dysbiosis of the gut microbiota, with overgrowth of pro-inflammatory or genotoxic bacteria, disruption of the mucosal barrier, and metabolic endotoxemia. This dysregulated microbiota–immune axis creates an immunosuppressive and pro-tumorigenic milieu that favors CRC initiation and metastatic spread (20, 21). Together, these mechanisms provide a plausible biological rationale for the substantial and heterogeneous burden of CRC attributable to high FPG observed across SDI regions and between sexes in our GBD-based analysis.

In addition to high FPG, metabolic dysfunction-associated steatotic liver disease (MASLD) is increasingly recognized as a key metabolic comorbidity that may influence colorectal carcinogenesis through insulin resistance, chronic low-grade inflammation, and disruption of the gut–liver axis. Recent cohort data suggest that liver steatosis and MASLD are more frequent in patients who subsequently develop CRC and that higher fasting plasma glucose and non-invasive liver fibrosis scores jointly identify individuals at particularly high risk (22, 23). Furthermore, an updated meta-analysis of cohort studies has shown that MASLD is associated with an increased risk of colorectal cancer and colorectal adenoma, with risk gradients according to MASLD severity (24). Mechanistically, MASLD is characterized by hepatic lipid accumulation, oxidative stress, systemic and portal inflammation, and gut microbiota dysbiosis, all of which may promote a pro-tumorigenic microenvironment in the colorectum (25). Lifestyle factors that drive both MASLD and CRC, such as Westernized dietary patterns and physical inactivity, also appear to be important upstream determinants (23). In our GBD-based analysis, MASLD was not included as a separate risk factor in the comparative risk assessment framework, and we could not evaluate its potential confounding or mediating role between high FPG and CRC burden.

This study found a significant sex imbalance in the disease burden of high FPG-related colorectal cancer, with a higher burden in males than in females. The gender preference for this type of metabolism-driven cancer may be due to multiple mechanisms. Androgens promote the progression of the colorectal adenoma-cancer sequence by enhancing serine phosphorylation of insulin receptor substrate-1 (IRS-1) (26), aggravating insulin resistance, and activating the Wnt/β-catenin signaling pathway (27), while estrogens form a protective barrier for women by inhibiting IGF-1 receptor dimerization (28) and upregulating the expression of DNA mismatch repair genes (such as MLH1) (29). Male-dominated visceral obesity causes adipocytes to release more free fatty acids and proinflammatory factors, such as IL-6, driving the glycolytic reprogramming of colorectal epithelial cells (30, 31), while higher adiponectin secretion in female subcutaneous fat has anti-inflammatory (32) and insulin-sensitizing effects (33). These mechanisms should be regarded as hypotheses rather than conclusions from the present analysis, as the GBD data do not include individual-level information on adiposity distribution or hormonal status. In contrast, there is direct empirical evidence that gendered behavior and health system factors contribute to sex differences in CRC outcomes. The blood glucose control achievement rate and CRC screening compliance of male diabetic patients are lower than those of female patients (34, 35), resulting in the dual risks of prolonged exposure to hyperglycemia and delayed diagnosis of tumor stage.

To reduce the global burden of colorectal cancer (CRC) related to high FPG, it is urgent to build an intervention system of “prevention-screening-treatment.” At the primary prevention level, fiscal levers and food policies should be used to reshape the metabolic environment of the population (36, 37), and targeted diabetes prevention and control education should be conducted for men and groups with low health literacy. Secondary prevention requires the integration of CRC screening into the diabetes management pathway, promoting low-cost fecal occult blood testing and mobile colonoscopy technology to overcome resource limitations (38, 39). Tertiary intervention should improve the accessibility of new hypoglycemic and anticancer drugs in low- and middle-income countries through tiered pricing and generic drug production (40, 41). Simultaneously, it is necessary to optimize the priority of resource allocation based on GBD dynamic monitoring data-high SDI regions focusing on precision screening technology innovation, and low SDI regions relying on international funds to strengthen basic metabolic monitoring facilities. Only through cross-sectoral policy coordination, technology popularization, and health equity reform can the rising trend of high FPG-derived CRC be curbed and the substantial reduction of the burden of non-communicable diseases in the United Nations Sustainable Development Goal (SDG 3.4) (42) be achieved.

## Limitations

This study has several limitations that need to be addressed. First, the cancer registration system on which the GBD database relies has insufficient coverage in low-income countries, which may lead to an underestimation of the disease burden. Second, our estimates inherit the attribution assumptions and limitations of the GBD comparative risk assessment framework. In the GBD, the burden of CRC attributable to high FPG is quantified under a counterfactual scenario in which population FPG levels are reduced to a theoretical minimum-risk exposure level, using relative risks derived from meta-analyses of prospective studies. Although these models were adjusted for major confounders, residual confounding from other correlated metabolic exposures, such as high BMI, high systolic blood pressure, and high low-density lipoprotein cholesterol, cannot be fully excluded. Moreover, metabolic risk factors tend to cluster, and despite the use of joint risk-attribution methods and mediation adjustments in the GBD, some overlap between the effects of high FPG levels and other metabolic abnormalities may persist. In addition, ecological analysis based on population-level data is difficult to completely exclude the interference of reverse causality on the risk effect size. Finally, the BAPC prediction model does not include possible breakthroughs in public health interventions or sudden global events, which may weaken the robustness of long-term predictions. In several comparisons, overlapping uncertainty intervals indicated that differences between regions/sexes/time periods may not reach statistical significance. Future studies should combine individual-level cohort data with metabolomics technology to further analyze the causal pathway of high FPG-derived CRC and the mechanism of sex/regional heterogeneity.

## Data Availability

The original contributions presented in the study are included in the article/**Supplementary Material**. Further inquiries can be directed to the corresponding author.

## Supplementary material

Supplementary Table 1 Global and Regional Population Attributable Fractions of Colon and Rectum Cancer Deaths and DALYs Due to High Fasting Plasma Glucose in 1990 and 2021.

Supplementary Table 2 The Slope Index of Inequality and the Concentration Index for Colon and Rectum Cancer Attributable to High Fasting Plasma Glucose from 1990 to 2021.

Supplementary Table 3 Projection of the Global Burden of Colon and Rectum Cancer Attributable to High Fasting Plasma Glucose for the Next 15 Years Based on the BAPC Model.
