# Supplementary material for: Global, regional and national burden of colon and rectum cancer attributable to high fasting plasma glucose: a systematic analysis for the Global Burden of Disease Study 2021
Source: Front Oncol. 2026 Feb 27;16:1690349. doi: 10.3389/fonc.2026.1690349 (PMC12982019; doi:10.3389/fonc.2026.1690349)
Supplement: Supplementary Table 1 — Global and Regional Population Attributable Fractions of Colon and Rectum Cancer Deaths and DALYs Due to High Fasting Plasma Glucose in 1990 and 2021. [file DataSheet1.docx]

**Table 1.** **Global and Regional Population Attributable Fractions of Colon and Rectum Cancer Deaths and DALYs Due to High Fasting Plasma Glucose in 1990 and 2021**

| **Location** | **Deaths** | | **DALYs** | |
| --- | --- | --- | --- | --- |
|  | **1990** | **2021** | **1990** | **2021** |
| **Global** | **14.29%** | **16.93%** | **13.32%** | **15.69%** |
| **Region** | | | | |
| **Andean Latin America** | **10.42%** | **14.18%** | **9.20%** | **13.13%** |
| **Australasia** | **11.15%** | **16.40%** | **9.94%** | **14.99%** |
| **Caribbean** | **13.24%** | **15.05%** | **12.14%** | **14.03%** |
| **Central Asia** | **11.79%** | **14.06%** | **10.46%** | **12.91%** |
| **Central Europe** | **16.35%** | **19.94%** | **15.15%** | **18.89%** |
| **Central Latin America** | **17.39%** | **21.51%** | **15.77%** | **20.37%** |
| **Central Sub-Saharan Africa** | **17.34%** | **17.10%** | **16.06%** | **15.88%** |
| **East Asia** | **15.31%** | **17.70%** | **15.06%** | **16.71%** |
| **Eastern Europe** | **9.52%** | **12.32%** | **8.77%** | **11.56%** |
| **Eastern Sub-Saharan Africa** | **4.95%** | **6.97%** | **4.32%** | **6.17%** |
| **High-income Asia Pacific** | **18.09%** | **20.29%** | **16.11%** | **18.74%** |
| **High-income North America** | **14.72%** | **18.09%** | **13.39%** | **16.82%** |
| **North Africa and Middle East** | **12.48%** | **19.31%** | **11.45%** | **18.15%** |
| **Oceania** | **13.41%** | **14.98%** | **12.63%** | **13.97%** |
| **South Asia** | **13.64%** | **17.96%** | **12.95%** | **17.21%** |
| **Southeast Asia** | **9.52%** | **11.93%** | **8.53%** | **10.85%** |
| **Southern Latin America** | **13.37%** | **14.76%** | **12.19%** | **13.82%** |
| **Southern Sub-Saharan Africa** | **10.69%** | **15.40%** | **9.75%** | **14.26%** |
| **Tropical Latin America** | **10.42%** | **18.31%** | **18.22%** | **17.48%** |
| **Western Europe** | **14.70%** | **16.72%** | **13.57%** | **15.47%** |
| **Western Sub-Saharan Africa** | **10.02%** | **14.17%** | **8.91%** | **12.57%** |

**Table 2. The Slope Index of Inequality and the Concentration Index for Colon and Rectum Cancer Attributable to High Fasting Plasma Glucose from 1990 to 2021.**

|  |  | **Deaths （95%CI）** | **DALYs (95%CI)** |
| --- | --- | --- | --- |
| **Slope Index of Inequality (95% CI)** | **1990** | **0.989(0.827,1.151)** | **20.251(16.933,23.569)** |
|  | **2021** | **0.996(0.821,1.172)** | **20.292(16.949,24.036)** |
| **Concentration Index (95% CI)** | **1990** | **-0.398(-0.468, -0.330)** | **-0.344(-0.417, -0.273)** |
|  | **2021** | **-0.339(-0.426, -0.255)** | **-0.294(-0.383, -0.206)** |

**DALYs Disability-Adjusted Life Years, CI confidence interval.**

**Table 3. Projection of the Global Burden of Colon and Rectum Cancer Attributable to High Fasting Plasma Glucose for the Next 15 Years Based on the BAPC Model.**

| **Year** | **Deaths (95%UI)** | | **DALYs (95%UI)** | |
| --- | --- | --- | --- | --- |
|  | **Absolute numbers** | **ASR (per 100,000)** | **Absolute numbers** | **ASR in (per 100,000)** |
| 2022 | 79679 (78068,81289) | 0.99 (0.97,1.01) | 1668205 (1630594,1705815) | 20.73 (20.27,21.2) |
| 2023 | 79956 (77480,82433) | 0.98 (0.95,1.01) | 1679499 (1623944,1735054) | 20.68 (19.99,21.36) |
| 2024 | 80221 (76647,83795) | 0.98 (0.94,1.02) | 1690352 (1611416,1769287) | 20.62 (19.65,21.58) |
| 2025 | 80475 (75627,85324) | 0.97 (0.91,1.03) | 1700817 (1594332,1807303) | 20.56 (19.27,21.85) |
| 2026 | 80712 (74434,86990) | 0.97 (0.89,1.04) | 1710586 (1572950,1848223) | 20.5 (18.85,22.15) |
| 2027 | 80944 (73092,88796) | 0.96 (0.87,1.05) | 1720311 (1548125,1892497) | 20.44 (18.39,22.49) |
| 2028 | 81171 (71620,90722) | 0.96 (0.84,1.07) | 1729911 (1520240,1939582) | 20.39 (17.92,22.86) |
| 2029 | 81386 (70023,92749) | 0.95 (0.82,1.08) | 1739113 (1489312,1988915) | 20.33 (17.41,23.25) |
| 2030 | 81595 (68314,94876) | 0.95 (0.79,1.1) | 1747767 (1455346,2040189) | 20.28 (16.89,23.67) |
| 2031 | 81792 (66487,97097) | 0.94 (0.77,1.12) | 1755517 (1418080,2092954) | 20.22 (16.33,24.1) |
| 2032 | 81980 (64546,99415) | 0.94 (0.74,1.14) | 1763144 (1378139,2148148) | 20.16 (15.76,24.56) |
| 2033 | 82157 (62497,101817) | 0.93 (0.71,1.16) | 1770843 (1335902,2205783) | 20.11 (15.17,25.05) |
| 2034 | 82320 (60347,104294) | 0.93 (0.68,1.18) | 1778176 (1291174,2265178) | 20.06 (14.57,25.55) |
| 2035 | 82477 (58104,106850) | 0.92 (0.65,1.2) | 1784696 (1243719,2325673) | 20.01 (13.94,26.07) |
| 2036 | 82622 (55764,109479) | 0.92 (0.62,1.22) | 1790058 (1193335,2386781) | 19.94 (13.29,26.59) |

**DALYs Disability-Adjusted Life Years, ASR Age-Standardized Rate, UI uncertainty interval.**
